# Identification of the Novel Candidate Genes and Variants in Boar Liver Tissues with Divergent Skatole Levels Using RNA Deep Sequencing

**DOI:** 10.1371/journal.pone.0072298

**Published:** 2013-08-26

**Authors:** Asep Gunawan, Sudeep Sahadevan, Mehmet Ulas Cinar, Christiane Neuhoff, Christine Große-Brinkhaus, Luc Frieden, Dawit Tesfaye, Ernst Tholen, Christian Looft, Dessie Salilew Wondim, Michael Hölker, Karl Schellander, Muhammad Jasim Uddin

**Affiliations:** 1 Institute of Animal Science, Faculty of Agriculture, University of Bonn, Bonn, Germany; 2 Department of Animal Production and Technology, Faculty of Animal Science, Bogor Agricultural University, Bogor, Indonesia; 3 Fraunhofer Institute for Algorithms and Scientific Computing (SCAI), Schloss Birlinghoven, Sankt Augustin, Germany; 4 Department of Animal Science, Faculty of Agriculture, Erciyes University, Kayseri, Turkey; University of Torino, Italy

## Abstract

Boar taint is the unpleasant odour of meat derived from non-castrated male pigs, caused by the accumulation of androstenone and skatole in fat. Skatole is a tryptophan metabolite produced by intestinal bacteria in gut and catabolised in liver. Since boar taint affects consumer’s preference, the aim of this study was to perform transcriptome profiling in liver of boars with divergent skatole levels in backfat by using RNA-Seq. The total number of reads produced for each liver sample ranged from 11.8 to 39.0 million. Approximately 448 genes were differentially regulated (p-adjusted <0.05). Among them, 383 genes were up-regulated in higher skatole group and 65 were down-regulated (p<0.01, FC>1.5). Differentially regulated genes in the high skatole liver samples were enriched in metabolic processes such as small molecule biochemistry, protein synthesis, lipid and amino acid metabolism. Pathway analysis identified the remodeling of epithelial adherens junction and TCA cycle as the most dominant pathways which may play important roles in skatole metabolism. Differential gene expression analysis identified candidate genes in ATP synthesis, cytochrome P450, keratin, phosphoglucomutase, isocitrate dehydrogenase and solute carrier family. Additionally, polymorphism and association analysis revealed that mutations in *ATP5B, KRT8, PGM1, SLC22A7* and *IDH1* genes could be potential markers for skatole levels in boars. Furthermore, expression analysis of exon usage of three genes (*ATP5B, KRT8* and *PGM1*) revealed significant differential expression of exons of these genes in different skatole levels. These polymorphisms and exon expression differences may have impacts on the gene activity ultimately leading to skatole variation and could be used as genetic marker for boar taint related traits. However, further validation is required to confirm the effect of these genetic markers in other pig populations in order to be used in genomic selection against boar taint in pig breeding programs.

## Introduction

Boar taint is the offensive odour or taste that can be evident during the cooking or eating of porcine meat derived from non-castrated male pigs. It is preliminary caused by the accumulation of androstenone and skatole in the adipose tissues. Skatole is a metabolite of tryptophan which is produced by intestinal bacteria such Clostridium and Bacteroides genera in gut and metabolised in the liver (reviewed by Wesoly and Weiler [1]). It has a fecal-like odor and unlike the smell of androstenone, the vast majority of people are able to detect the smell of skatole. It is well known that high concentrations of male sex steroids such as androstenone prevent the enzymes responsible for skatole metabolism resulting in the reduction of skatole metabolism in liver and accumulation in adipose tissue [2]. Notably, the most common practice to prevent this smell is the castration of male piglets. But castration is undesirable due to ethical and economical concerns [3], [4] and castration of piglets is announced to be banned in the European Community by 2018 [5] creating an urgent need to develop alternative methods to prevent tainted meat.

In pigs, skatole is absorbed by the intestinal mucosa into the portal vein and passes through the liver where it is efficiently metabolised. Three major metabolites of skatole isolated from pigs are 6-sulfatoxyskatole (MII), 3-hydroxy-3-methyloxindole (MIII) and 3-methyl indole [6]. Among these skatole metabolites, MII is secreted in plasma and urine as a sulphate conjugate, and MIII is found to be related to the skatole levels in fat [6]. It has been demonstrated that the liver has a potential capacity to extract skatole from blood [7]. However, in boars a proportion of skatole passes the liver without being metabolised and accumulates in the adipose tissue that is responsible for tainted meat [6]. With the aim to identify candidate genes, a number of quantitative trait loci (QTL) analysis have been conducted for skatole in purebred and crossbred pig populations [8], [9]. Several QTL for skatole were identified on different pig chromosomes such as on SSC6, SSC7, SSC12, SSC13, SSC14 and SSCX in different pig populations [9], [10]. A few studies performed polymorphism and association analysis of selected genes [11], [12] and a study was devoted to perform a genome wide association [13] for skatole in pigs. In this regard, the genes coding for enzymes of the cytochrome family received considerable interest due to their role in skatole metabolism, such as cytochrome P4502E1 (CYP2E1) is the main hepatic enzyme involved in the metabolism of skatole in the liver [14]. Significant associations have been identified for the polymorphism of CYP2E1 and CYP21 genes, and these genes are reported to reduce skatole levels in pigs [7]. A mutation in the coding region of CYP2A6 was found to be associated with high level skatole in fat [15].

Several studies are devoted to indentify the genes and pathways involved in the androstenone metabolism in liver [16], [17], [18] but to the authors’ knowledge, no study was devoted to perform a global transcriptome analysis for divergent skatole levels in boar fat as well as to identify the pathways that might be involved in skatole metabolism in liver. RNA-Seq is a recently developed next generation sequencing technology for transcriptome profiling that boosts identification of novel and low abundant transcripts [19]. It could be used to analyse changes in gene expression across the entire transcriptome [19], [20]. RNA-Seq also provides evidence for identification of splicing events, polymorphisms and different family isoforms of transcripts [21]. Therefore, the major aim of this study was to elucidate the genes and pathways involved in skatole metabolism in liver tissue using RNA deep sequencing technology. For this purpose, we performed differential expression analysis of genes in liver samples from boars with high skatole (HS) and low skatole (LS) in their backfat. Additionally, gene polymorphism analysis and differential exon usage analysis were also performed for the differentially expressed genes.

## Results

### Analysis of RNA Deep Sequencing Data

We sequenced cDNA libraries from 6 samples from liver tissues (3 from HS in backfat and 3 from LS in backfat) using Illumina HiSeq 2000 as a part of our previous work [16]. The details of the sequencing are mentioned by Gunawan et al [16] and the raw sequencing data is deposited in GEO database and available under the accession id GSE44171. The sequencing produced clusters of sequence reads with maximum of 100 base-pair (bp). After quality control and filtering, the total number of reads for liver samples ranged from 11.8 to 39.0 million with a median of 22.8 million. Total number of reads for each group of liver sample and the number of reads mapped to reference sequences are shown in Table 1. In case of liver from LS group 43% to 74.4% of the total reads were aligned to reference sequence whereas, in case of liver from the HS group 61.3% to 84% were aligned.

**Table 1:** Summary of sequence read alignments to reference genome in liver samples.

Group	Sample*	Total number of reads before QC(million)	Total number of reads after QC(million)	Un-mapped reads (%)	Mapped reads (%)
Low skatole	LS1	29.5	23.4	6 (25.6)	17.4 (74.4)
	LS2	46	35.6	10 (28)	25.6 (72)
	LS3	14.6	12.6	7.2 (57)	5.4 (43)
High skatole	HS1	16.4	14.7	5.7 (38.7)	9 (61.3)
	HS2	13.3	11.8	4.9 (41.5)	6.9 (58.5)
	HS3	45.2	39	6.3 (16)	32.7 (84)

* LS 1, 2, 3 indicate the low skatole sample; HS 1, 2, 3 indicate the high skatole samples.

### Differential Gene Expression Analysis

**Figure 1:**
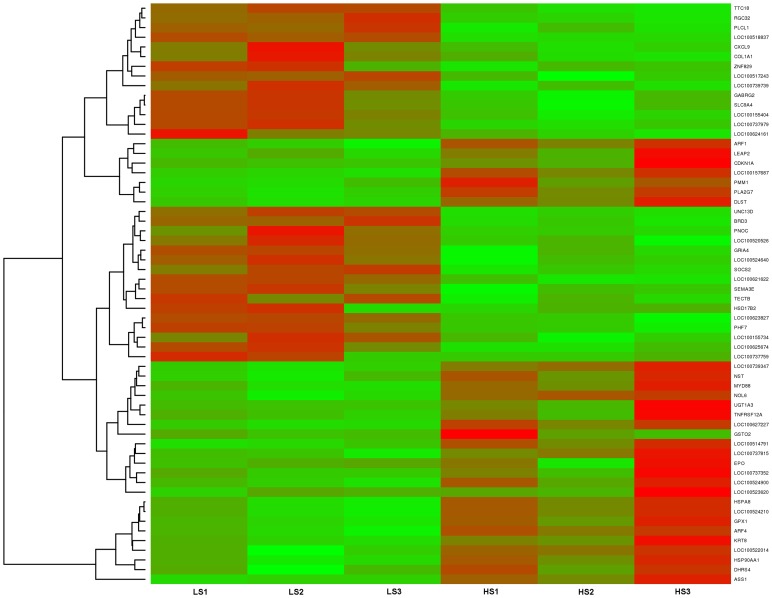
Heatmap showing differentially expressed genes in liver samples. The red blocks represent over expressed genes, and the green blocks represent under expressed genes. Legend: LS1–LS3 boars liver with low skatole in backfat and HS1–HS3 boars liver with high skatole in backfat.

Differential gene expression from livers of boars with HS and LS levels in backfat were calculated from the raw reads using the R package DESeq [22]. The significance scores were corrected for multiple testing using Benjamini-Hochberg correction. We used a negative binomial distribution based method implemented in DESeq to identify differentially expressed genes (DEGs) in the liver with divergent (HS and LS in backfat) skatole levels. A total of 448 DEGs were selected from the differential expression analysis using the criteria *p*_adjusted_<0.05 and log2 fold change>1.5 (Table S1). In the liver tissues, 383 genes were found to be highly expressed in high skatole group whereas, 65 genes were found to be highly expressed in low skatole group (Table S1). The range of log2 fold change values for DEGs was from −6.79 to 5.82. Heatmaps (Figure 1) illustrate the top 30 up and top 30 down regulated genes identified in the liver tissues from HS and LS boars. The top 30 up and down regulated genes identified in the liver tissues with different skatole levels along with log FC and *p* values are listed in Table 2. The differential expression analysis of our data revealed both novel transcripts and common genes which were previously identified in various gene expression studies. The novel transcripts from our analysis and commonly found genes are mentioned in detail in the discussion section.

**Table 2:** Top 30 up and down regulated genes in liver tissues collected from boars with high and low skatole levels in backfat.

Gene	Orthologue gene description	Reference ID	log2Fold Change	*p*-adj.
SERPINA12	Serpin peptidase inhibitor, clade A (alpha-1 antiproteinase, antitrypsin),member 12	XM_003128704.2	5.82	4.73e-06
KRT8	Keratin 8	NM_001159615.1	4.33	6.03e-05
TNFRSF12A	Tumor necrosis factor receptor superfamily, member 12A	NM_001142839.1	3.75	2.05e-09
HSPA8	Heat shock 70 kDa protein 8	NM_001243907.1	3.70	0.004
HSP71	Heat shock 70 kDa protein 8	XM_003129961.3	3.70	0.004
GSTO2	Glutathione S-transferase omega 2	XM_001927288.3	3.56	2.55e-07
CDKN1A	Cyclin-dependent kinase inhibitor 1A (p21, Cip1)	XM_001929558.1	3.44	1.95e-12
HSP90AA1	Heat shock protein 90 kDa alpha (cytosolic), class A member 1	NM_213973.1	3.21	0.0027
ALDH3B1	Aldehyde dehydrogenase 3 family, member B1	XM_003480660.1	3.15	4.06e-08
DLST	Dihydrolipoamide S-succinyltransferase (E2 component of 2-oxo-glutarate complex)	NM_214397.1	3.11	3.30e-10
UGT1A3	UDP glucuronosyltransferase 1 family, polypeptide A3	XM_003133742.3	3.06	3.55e-07
DHRS4	Dehydrogenase/reductase (SDR family) member 4	NM_214019.1	3.02	0.0039
SLC5A6	Solute carrier family 5 (sodium-dependent vitamin transporter), member 6	XM_003125293.3	2.97	0.0035
PMM1	Phosphomannomutase 1	NM_001184895.1	2.90	1.56e-09
NKIRAS2	NFKB inhibitor interacting Ras-like protein 2	XM_003358038.1	2.89	5.60e-05
FOS	FBJ murine osteosarcoma viral oncogene homolog	NM_001123113.1	2.86	3.21e-08
CYP4A25	Cytochrome P450 4A24	XM_003128016.3	2.81	2.81e-08
CYP4A24	Cytochrome P450 4A24	XM_003356476.2	2.76	2.03e-05
ATP5B	ATP synthase, H+ transporting, mitochondrial F1 complex, beta polypeptide	XM_001929410.2	2.74	0.00012
TUBA1A	Tubulin, alpha 1a	XM_003355375.1	2.71	1.50e-07
SLC25A5	Solute carrier family 25 (mitochondrial carrier; adenine nucleotidetranslocator), member 5	XM_001927440.2	2.71	0.00104
ATP5A1	ATP synthase, H+ transporting, mitochondrial F1 complex, alpha subunit 1, cardiac muscle	NM_001185142.1	2.66	2.88e-05
SDHD	Succinate dehydrogenase complex, subunit D, integral membrane protein	NM_001097516.1	2.66	3.34e-07
CRYAB	Crystallin, alpha B	XM_003357294.1	2.62	3.21e-06
UQCRFS1	Ubiquinol-cytochrome c reductase, Rieske iron-sulfur polypeptide 1	XM_003127002.1	2.63	4.62e-06
PGM1	Phosphoglucomutase 1	XM_003127945.2	2.60	6.22e-06
SLC22A7	Solute carrier family 22 (organic anion transporter), member 7	NM_001044617.1	2.60	2.81e-08
CYP4B24	Cytochrome P450 4B24	XM_003482090.1	2.58	2.35e-06
SLC25A25	Solute carrier family 25 (mitochondrial carrier; phosphate carrier),member 25	NM_001164510.1	2.53	1.351e-05
COX5A	Cytochrome c oxidase subunit Va	XM_003482239.1	2.47	0.00031
PRDX1	Peroxiredoxin 1	XM_003128040.1	2.47	8.22e-05
ACSL5	Acyl-CoA synthetase long-chain family member 5	XM_003359369.1	2.38	0.0001684
MDH2	Malate dehydrogenase 2, NAD (mitochondrial)	NM_001244153.1	2.34	5.91e-05
MDH1	Malate dehydrogenase 1, NAD (soluble)	NM_213874.1	2.30	5.89e-06
TUBA1B	Tubulin, alpha 1b	NM_001044544.1	2.16	0.00018
IDH1	Isocitrate dehydrogenase 1 (NADP+), soluble	XM_003483721.1	2.14	0.00043
DHRS1	Dehydrogenase/reductase (SDR family) member 1	XM_003128543.1	2.11	3.02e-05
HSPA5	Heat shock 70 kDa protein 5 (glucose-regulated protein, 78 kDa)	XM_001927795.4	2.11	0.0037
PGM3	Phosphoglucomutase 3	XM_001924419.2	2.05	9.56e-06
SLC25A1	Solute carrier family 25 (mitochondrial carrier; citrate transporter), member 1	NM_001190189.1	2.03	0.0022
GSTM2	Glutathione S-transferase mu 2	NM_001078684.1	2.02	0.0017
TNFAIP1	Tumor necrosis factor, alpha-induced protein 1 (endothelial)	XM_003483067.1	1.91	0.00066
HSD3B7	Hydroxy-delta-5-steroid dehydrogenase, 3 beta- and steroiddelta-isomerase 7	XM_003124487.1	1.84	0.0012
SEC13	SEC13 homolog (S, cerevisiae)	XM_003483983.1	−1.93	0.0022
ZNF238	Zinc finger protein 238	XM_003357648.1	−1.93	0.0029
HDAC9	Histone deacetylase 9	XM_003357464.1	−1.94	0.0017
CEBPA	CCAAT/enhancer binding protein (C/EBP), alpha	XM_003127015.1	−1.94	0.0009
GUCY1A2	Guanylate cyclase 1, soluble, alpha 2	XM_003130093.3	−2.00	0.0036
LAMP1	Lysosomal-associated membrane protein 1	NM_001011507.1	−2.10	0.00029
SEMA3E	Sema domain, immunoglobulin domain (Ig), short basic domain, secreted, (semaphorin) 3E	XM_003130220.3	−2.37	0.0044
ZNF829	Zinc finger protein 829	XM_003127093.3	−2.40	0.00257
GABARAPL1	GABA(A) receptor-associated protein like 1	XM_003126479.3	−2.46	8.44e-05
UNC13D	Unc-13 homolog D (C, elegans)	XM_003131192.1	−2.54	0.00021
HSD17B2	Hydroxysteroid (17-beta) dehydrogenase 2	NM_001167649.1	−2.78	0.0032
CXCL9	Chemokine (C-X-C motif) ligand 9	NM_001114289.2	−2.80	0.0002
PNOC	Prepronociceptin	NM_001244476.1	−2.81	0.00085
CDK5	Cyclin-dependent kinase 5	XM_003480595.1	−2.82	0.00017
PHF7	PHD finger protein 7	XM_001928213.2	−2.83	0.00345
SOCS2	Suppressor of cytokine signaling 2	NM_001097461.1	−2.96	0.00054
PCLO	Piccolo presynaptic cytomatrix protein	XM_003357489.2	−2.97	0.0006
SLC9A4	Solute carrier family 9 (sodium/hydrogen exchanger), member 4	XM_003354711.1	−3.36	0.0006
GABRG2	Gamma-aminobutyric acid (GABA) A receptor, gamma 2	XM_003359825.1	−3.40	0.0006
LOC100737161	LOC100737161	XM_003482919.1	−3.50	0.0001
LOC100512296	LOC100512296	XM_003129119.2	−4.07	1.01e-07
LOC100155734	LOC100155734	XM_001927727.2	−4.27	0.002
LOC100739739	LOC100739739	XM_003482938.1	−5.02	0.0011
LOC100625674	LOC100625674	XM_003359731.2	−5.89	0.0012
LOC100737759	LOC100737759	XM_003482870.1	−6.80	7.76e-05

### Biological Function Analysis for DEGs

**Figure 2:**
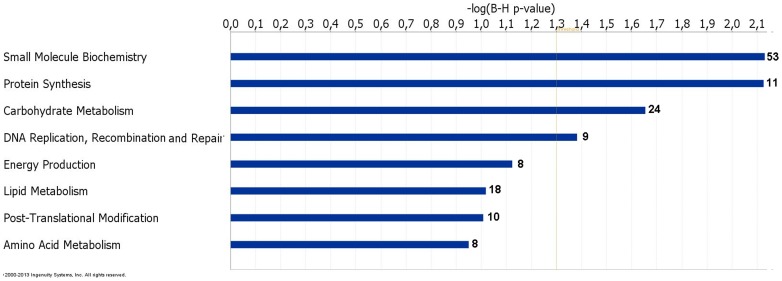
Functional grouping of DEGs in liver from boars with high and low skatole using Ingenuity Pathways Analysis (IPA) software. The most significant functional groups (*p*<0.05) are presented graphically. The bars represent the *p*-value on a logarithmic scale for each functional group.

**Figure 3:**
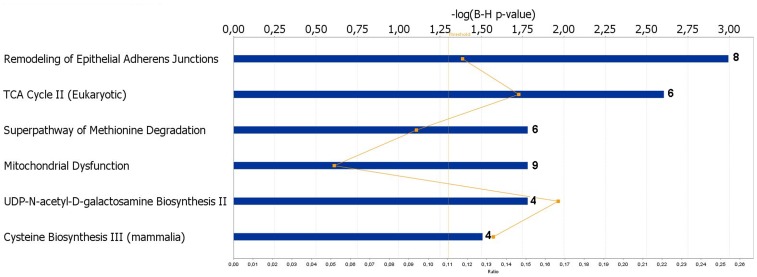
Canonical pathways of DEGs in liver from boars with high and low skatole using Ingenuity Pathways Analysis software. The most significant functional groups (*p* < 0.05) are presented graphically. The bars represent the *p*-value on a logarithmic scale for each functional group.

To investigate gene functions and to uncover the common processes and pathways among the selected DEGs, Ingenuity Pathway Analysis (IPA) software (Ingenuity Systems, www.ingenuity.com) was used. In the liver samples, out of 448 DEGs, 300 were assigned to a specific functional group based on the information from IPA (Figure 2). A large proportion (67.0%) of the DEGs from liver tissues in the high skatole group belong to the Gene Ontology (GO) category metabolic processes. The enriched GO metabolic processes include small molecule biochemistry, protein synthesis, carbohydrate metabolism, DNA replication, recombinant and repair, energy production and lipid metabolism. Other enriched GO categories include post translation modification and amino acid metabolism. The genes classified into each functional group are listed in the Table 3. IPA assigned 68 DEGs between high and low skatole group liver samples to six different canonical pathways. Canonical pathway analysis identified remodelling of epithelial adherens junctions and TCA cycle as the dominant pathways which play regulatory roles in the metabolic pathways (Figure 3). Other pathway categories including super pathways of methionine degradation, mitochondrial dysfunction, UDP-N-acetyl-D-galactosamine biosynthesis and cystein were also enriched (Figure 3). The genes assigned to these pathways in the liver with high and low skatole levels are presented in Table 4.

**Table 3:** Functional categories and corresponding genes those were over expressed in liver from high skatole boars.

Function	Numberof genes	B-H p-value	Genes
Small molecule biochemistry	53	7.41E-03-1.46E-01	DLST, IDH3B, MDH1, MDH2, ATP5A1, ATP5D, HSP90AA1, HSPA8, TAP1, CRYAB, GPI, NUDT15, AGXT, CTH, GNMT, GOT1, HAL, CTPS1, SLC25A5, CD1D, LBP, ASS1, BCKDHA, PEPD, GPX4, PRDX1, ACSL5, HAL, ABHD5, ARF1, FOS, MYD88, PISD, SERINC2, ANXA1, CIDEC, GOS2, PLA2G7, AP2M1, ACOX1, GPR39, GUCY1A2, GALE, CDKN1A, GUCY1A1, LT4N, PLOD1, POFUT1, PGM1, PMM1, PGM3, DNPEP, SLC22A7
Protein synthesis	11	7.41E-03-1.46E-01	CTH, GPX1, GPX4, GSTM2, IDH1, SOD2, CD1D, HSPA5, GNMT, DNPEP, DHRS4
Carbohydrate metabolism	24	2.21E-02-1.46E-01	NUDT15, TAP1, CEBPA, CDKN1A, HSP90AA1, HSPA8, IDH1, IDH3B, CRYAB, GALE, GPI, PGM1, PGM3, PMM1, AP2M1, CD1D, LBP, GOT1, ABHD5, PLA2G7, FOS, MYD88, GSTO2, GNPNAT1
DNA replication. recombination and repairs	9	4.10E-04-1.85E-02	ATP5A1, ATP5B, ATP5D, HSP90AA1, NUDT15, HSPA8, CEBPA, CDKN1A, HSPA5
Energy production	8	1.72E-03-2.52E-02	ATP5A1, ATP5B, ATP5D, HSP90AA1, HSPA8, TAP1, MDH1, MDH2
Lipid metabolism	18	9.59E-02-1.46E-02	MDH1, MDH2, CD1D, LBP, ABHD5, ACSL5, ARF1, FOS, GPX4, MYD88, PISD, CIDEC, PLA2G7, AP2M1, ACOX1, HSPA8, SAT1, SLC22A7
Post-tralslation modification	10	9.82E-02-1.46E-01	CRYAB, HSP90AA1, HSPA5, HSPA8, PDIA6, CTH, GNMT, SOD2, PLOD1, DHRS4
Amino acid metabolism	8	7.52E-02-9.59E-02	AGXT, CTH, GNMT, GOT1, ASS1, GNMT, GPX4, CDKN1A

**Table 4:** The canonical pathways from the IPA knowledge base that involve transcripts over expressed in liver from higher skatole boars.

Category	−log (B-Hp-value)	Number ofgenes	B-H- p-value	Genes
Remodeling of epithelial adherens junction	2.99	8	1.01E-03	ACTB, ARPC3, ARPC1A, TUBA1A1, TUBA1B, TUBA1C, TUBB2A, TUBB4B
TCA cycle	2.60	6	2.48E-03	DLST, IDH3B, MDH1, MDH2, OGDHL, SDHD
Superpathway of methionine degradation	1.78	6	1.66E-02	AHCY, CTH, FTSJ1, GOT1, MUT, PRMT1,
Mithochondria1 dysfunction	1.78	9	1.66E-02	ATP5A1, ATP5B, COX5A, GPX4, NDUFA9, PSEN2, SDHD, SOD2, UQCRFS1
UDP-N-acety1-D-ga1actosamine biosynthesis II	1.78	4	1.66E-02	GALE, GNPNAT1, GPI, PGM3
Cysteine biosynthesis	1.50	4	3.11E-02	AHCY, CTH, FTSJ1, PRMT1

### Validation of Selected DEGs with Quantitative Real Time PCR (qRT-PCR)

**Figure 4:**
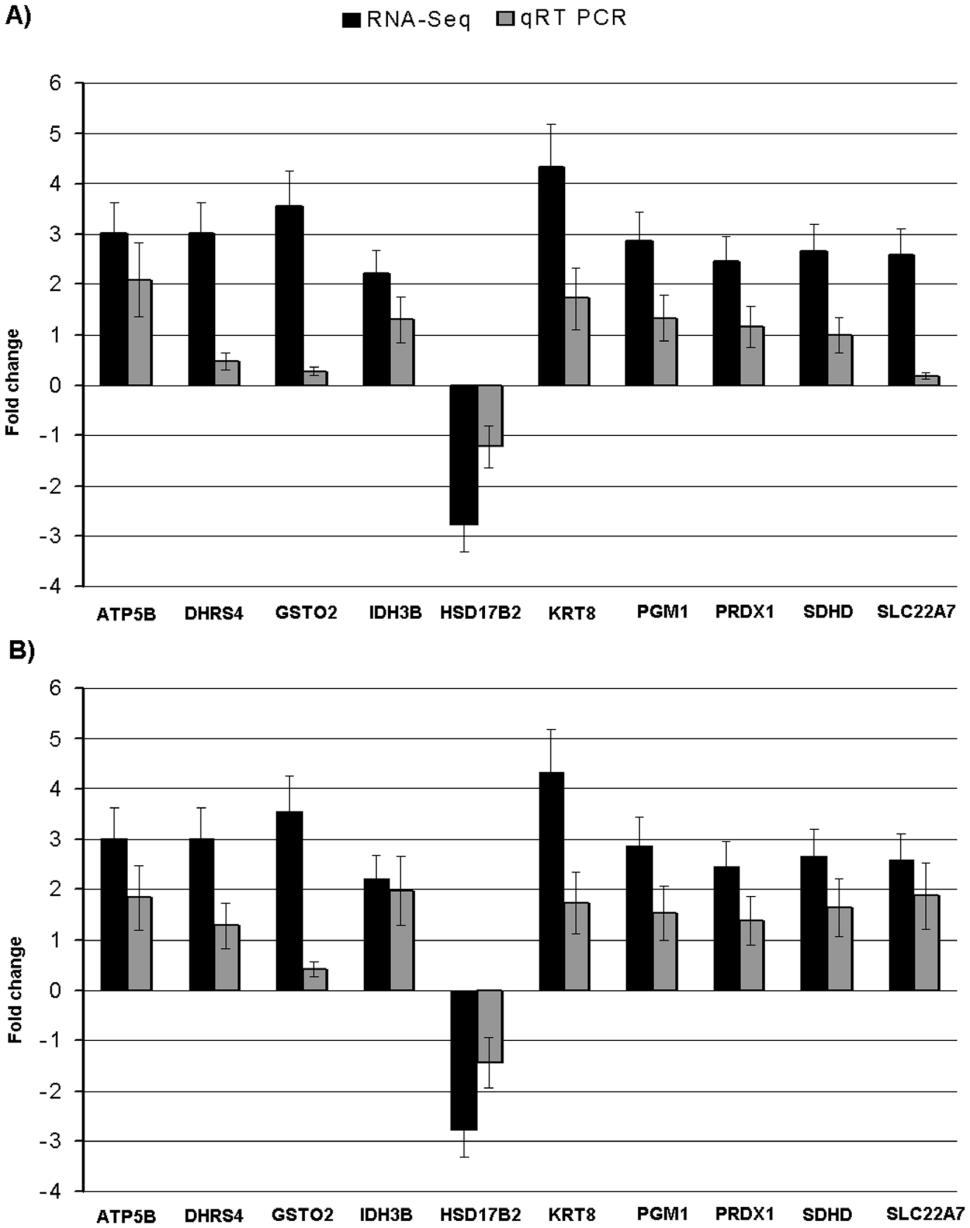
qRT-PCR validations for ten DEGs in liver from boars with divergent skatole levels. The validation was performed using the same RNA samples as used in the RNA deep sequencing (A); new group of boars with divergent skatole levels were created from the remaining 94 boars for the validation of the same DEGs using qRT-PCR (B). Fold change determined via division of high skatole group gene expression value by low skatole group gene expression value.

In order to validate the RNA-Seq results, on the basis of differential expressions and functions related to skatole, a total of 10 genes (*ATP5B*, *DHRS4*, *GSTO2*, *IDH3B*, *HSD17B2*, *KRT8*, *PGM1*, *PRDX1*, *SDHD* and *SLC22A7*) were selected and quantified using qRT-PCR. For this purpose, the same samples as used in the deep sequencing were used. Comparison of qRT-PCR data for 10 selected genes showed qualitative concordance of the expression with the RNA-Seq results (Figure 4A). To further validate the expression of selected genes more robustly, new grouping of independently high (n = 3) and low (n = 3) skatole were done among the remaining 94 pigs. The mRNA expressions of selected genes also showed similar pattern of expression in this new groups (Figure 4B). Gene expression values for qRT-PCR were normalized using housekeeping genes *PPIA* and *GAPDH* [23].

### Differential Exon Usages Analysis

**Figure 5:**
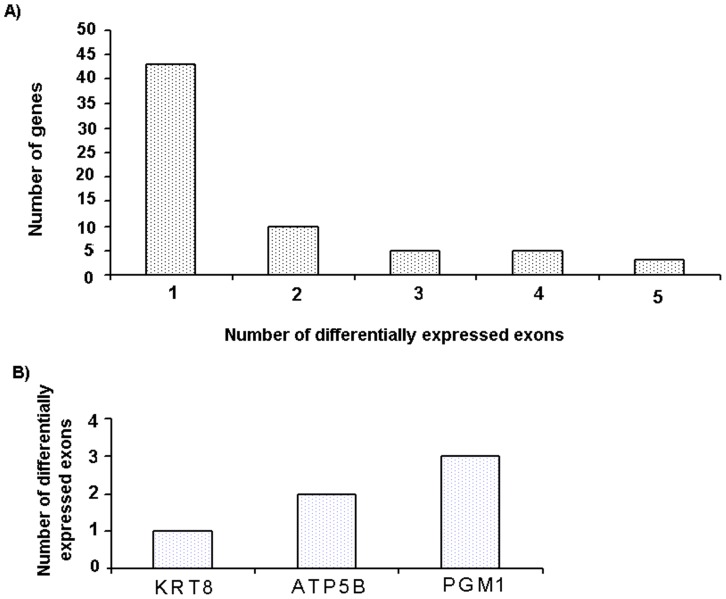
Distribution of the number of alternate splicing. The distribution of the number of alternate splicing the DEGs (A); number of alternate splicing in the selected genes (B).

**Figure 6:**
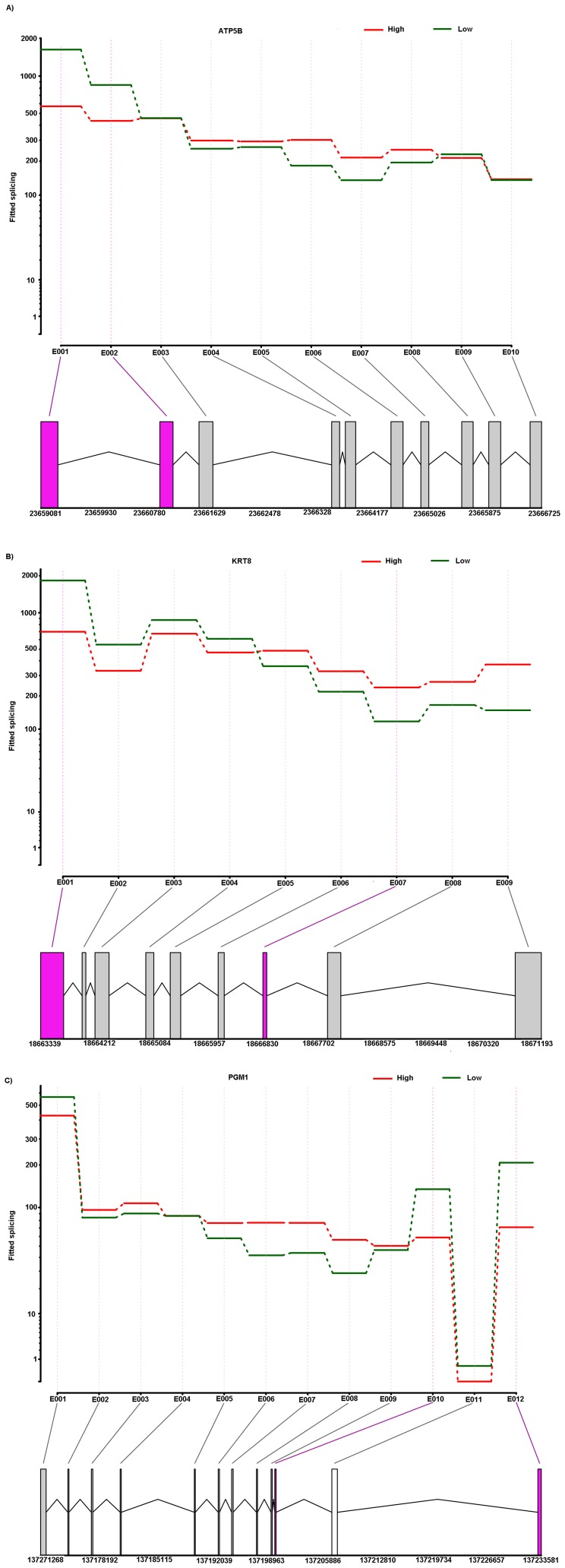
The schematic diagram of differential exon expression in selected genes. Differential exon expression in ATP5B (A). KRT8 (B) and PGM1(C). (Top panel) Fitted values according to the linear model; (middle panel) normalized counts for each sample; (bottom panel) flattened gene model. (Red) Data for high skatole samples; (green) low skatole.

In order to explore the expression pattern of different exons of a gene between HS and LS boars, selected DEGs were analysed for differential exon expression [24]. Distribution of differential exon events and selected differential exon usage genes are shown in figure 5A and 5B, respectively. We identified 126 (P-adj<0.05) differential exon expressions in 66 DEGs (Table S2) that showed differential usage of exons between high and low skatole. Selected differential exon usage identified in DEGs for liver samples are given in Table 5. It is important to note that some genes showed more than one variable splicing. We found that about 34.8% of the alternative spliced genes underwent multiple differential exon usage events (Figure 5A), illustrating the complexity of porcine transcriptome. Figure 6A, 6B and 6C show an example of differential exon expression for three genes (*ATP5B*, *KRT8* and *PGM1*) which were shown above to be associated with the skatole level. The first and second exon of the ATP5B gene showed significantly higher expressions in the low skatole (Figure 6A) group. The first and fifth exon of the KRT8 gene showed significantly higher expression levels in the low skatole than in high skatole group (Fig. 6B). Figure 6C showed that the 12^th^ exon of the PGM1 gene was expressed significantly higher in the LS than in HS group of boars.

**Table 5:** Differential exon expression in selected DEGs in liver samples from boars with divergent skatole levels in backfat.

Chr	GeneID (+/−indicates strand)	Transcripts	Gene Name	Exon ID	Start	End	Log2fold(high/low)	*P*-adjust
1	407060-	XM_001927795.4	HSPA5	E001	299754033	299754610	−0.175	0.049
3	100525281+	XM_003124487.1	HSD3B7	E007	17843326	17844524	−0.689	3.89e-05
4	100156038+	XM_003355245.1	PGM3	E010	119097423	119098520	−0.341	0.013
5	100157156−	XM_001929410.2	ATP5B	E001	23659081	23659341	−0.321	0.026
5	100157156−	XM_001929410.2	ATP5B	E002	23660897	23661098	−0.224	0.039
5	100152077−	NM_001159615.1	KRT8	E001	18663339	18663700	−0.286	0.001
5	100151790+	NM_001184895.1	PMM1	E008	4347163	4347697	−0.354	0.013
5	100151790+	NM_001184895.1	PMM1	E008	4347163	4347697	−0.354	0.013
6	397566−	XM_003127946.1	PGM1	E010	137200412	137200574	−0.417	0.013
6	397566−	XM_003127945.2	PGM1	E010	137200412	137200574	−0.417	0.013
6	397566−	XM_003127945.2	PGM1	E012	137233155	137233581	−0.504	3.35e-05
6	100512476+	XM_003128039.1	PRDX1	E003	153249744	153249811	−2.125	0.001
6	100621392+	XM_003356202.1	GALE	E011	75420510	75420891	−0.393	0.001
12	100737417+	XM_003483067.1	TNFAIP1	E007	46536059	46537702	0.222	0.040

### Gene Variation Analysis

**Figure 7:**
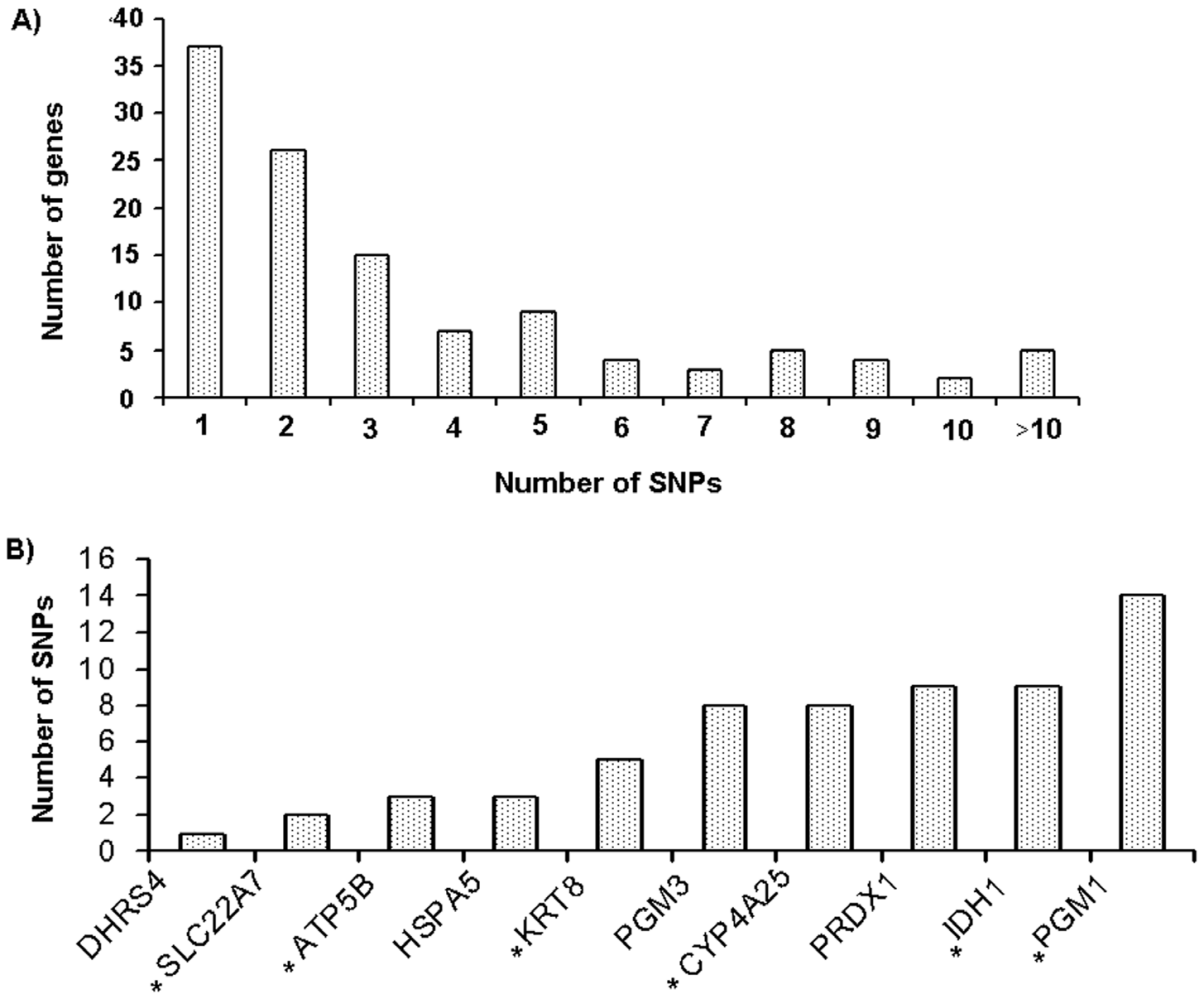
Distribution of the number of SNPs detected in the DEGs. The distribution of the number of SNPs occurred in each gene (A); numbers of SNPs in the genes selected for the association validation (B). *indicate the genes selected for the SNPs validation.

In the liver samples, 427 gene polymorphisms were identified in 107 DEGs (Table S3). Selected polymorphisms identified in DEGs for liver samples are given in Table 6. The distribution of SNPs number and selected SNPs used for validation is shown in figure 7A and 7B, respectively. We found that about 68.4% of genes had multiple polymorphisms (Figure 7A). Read counts for individual samples for identified polymorphisms in liver tissues are given in Table S4. In order to validate the SNP results, on the basis of functional SNP and functions related to skatole, a total of 6 SNP were selected for association study (Figure 7B and Table S5). We have selected SNPs in *ATP5B, KRT8, PGM1, CYP4A25, SLC22A7* and *IDH1* to validate their segregation (Table S5) and association in our population (n = 100). Out of 6 SNP, five SNPs were found to be associated with skatole levels in our (n = 100) population (Table S5). Polymorphisms in *ATP5B* (g.23661024 T>C), *KRT8* (g.18670859>A), *PGM1* (g.137174784C>A), *SLC22A7* (g.43833000 G>A) and *IDH1* (g.122862530 C>T) were associated with skatole level (Table 7).

**Table 6:** Polymorphisms detected in highly polymorphic DEGs.

Refseq ID	Gene name	Chr	Position	db SNP	Ref	Alt	High skatole coverage	High skatole mean phred score	Low skatole coverage	Low skatole mean phred score	Sample group	SNP clasification
XM 001929410.2	ATP5B	5	23659117	0	A	G	175	49	182	50	High and Low	Non Synonymous
XM 001929410.2	ATP5B	5	23661024	0	T	C	532	47	366	47	High and Low	Non Synonymous
XM 001929410.2	ATP5B	5	23661627	rs80908111	C	T	481	48	253	47	High and Low	Non Synonymous
NM 214019.1	DHRS4	7	80515824	0	T	C	0	0	162	49	Low skatole	Non Synonymous
NM 001159615.1	KRT8	5	18663991	0	T	C	0	0	343	50	Low skatole	Synonymous
NM 001159615.1	KRT8	5	18664266	0	T	C	515	49	317	48	High and Low	Non Synonymous
NM 001159615.1	KRT8	5	18664389	0	A	G	513	49	286	49	High and Low	Synonymous
NM 001159615.1	KRT8	5	18667998	0	G	A	328	49	0	0	High skatole	Synonymous
NM 001159615.1	KRT8	5	18670859	0	G	A	303	50	0	0	High skatole	Synonymous
XM 003128016.3	CYP4A25	6	152197351	0	A	C	251	49	0	0	High skatole	Non Synonymous
XM 003128016.3	CYP4A25	6	152198727	0	T	C	210	48	0	0	High skatole	Synonymous
XM 003128016.3	CYP4A25	6	152206224	0	A	G	279	48	0	0	High skatole	Synonymous
XM 003128016.3	CYP4A25	6	152206341	0	G	C	308	46	188	49	High and Low	Synonymous
XM 003128016.3	CYP4A25	6	152206385	0	A	T	184	45	172	49	High and Low	Synonymous
XM 003128016.3	CYP4A25	6	152206818	0	C	T	0	0	253	49	Low skatole	Non Synonymous
XM 003127945.2	PGM1	6	137171304	0	G	T	134	50	0	0	High skatole	Non Synonymous
XM 003127945.3	PGM1	6	137171323	0	T	C	204	50	0	0	High skatole	Synonymous
XM 003127945.4	PGM1	6	137171408	0	T	C	435	50	254	49	High and Low	Non Synonymous
XM 003127945.5	PGM1	6	137171452	0	A	G	403	50	0	0	High skatole	Non Synonymous
XM 003127945.6	PGM1	6	137171481	0	C	A	0	0	200	49	Low skatole	Non Synonymous
XM 003127945.7	PGM1	6	137171535	0	T	C	389	50	175	49	High and Low	Synonymous
XM 003127945.8	PGM1	6	137171741	0	A	C	383	50	145	50	High and Low	Synonymous
XM 003127945.9	PGM1	6	137171813	0	C	T	383	49	0	0	High skatole	Non Synonymous
XM 003127945.10	PGM1	6	137171857	0	C	T	380	49	123	50	High and Low	Non Synonymous
XM 003127945.11	PGM1	6	137174682	0	A	G	332	47	0	0	High skatole	Synonymous
XM 003127945.12	PGM1	6	137174727	0	C	T	294	45	0	0	High skatole	Synonymous
XM 003127945.13	PGM1	6	137174784	0	C	A	327	47	0	0	High skatole	Synonymous
XM 003127945.14	PGM1	6	137195153	0	A	G	256	49	0	0	High skatole	Synonymous
XM 003128039.1	PRDX1	6	153255729	rs81215265	C	T	451	48	236	48	High and Low	Non Synonymous
XM 003128039.3	PRDX1	6	153257939	rs81215269	C	T	0	0	242	45	low skatole	Synonymous
XM 003128039.4	PRDX1	6	153257940	rs81215270	C	G	448	48	242	45	High and Low	Synonymous
XM 003128039.5	PRDX1	6	153265783	0	G	A	513	48	365	46	High and Low	Synonymous
XM 003128039.6	PRDX1	6	153265829	rs196949554	G	T	475	48	338	46	High and Low	Synonymous
XM 003128039.7	PRDX1	6	153265881	0	A	G	307	49	187	47	High and Low	Synonymous
XM 003128039.9	PRDX1	6	153265905	0	T	C	201	49	0	0	High skatole	Synonymous
XM 003128039.10	SLC22A7	7	43833000	0	G	A	182	48	0	0	High skatole	Non Synonymous
XM 003128039.11	SLC22A7	7	43833898	0	G	A	197	49	0	0	High skatole	Synonymous
XM 003483721	IDH1	15	122861718	0	T	G	225	49	124	48	High and Low	Synonymous
XM 003483722	IDH1	15	122861896	0	C	T	430	48	235	48	High and Low	Synonymous
XM 003483723	IDH1	15	122861903	0	CGGG	CGG	424	48	233	48	High and Low	Deletion
XM 003483724	IDH1	15	122861968	0	C	T	441	49	223	49	High and Low	Non Synonymous
XM 003483725	IDH1	15	122862291	0	T	C	374	49	177	49	High and Low	Synonymous
XM 003483727	IDH1	15	122862513	0	C	T	406	49	133	48	High and Low	Synonymous
XM 003483728	IDH1	15	122862530	0	C	T	397	48	118	48	High and Low	Synonymous
XM 003483729	IDH1	15	122876927	0	G	A	292	49	0	0	High skatole	Synonymous

**Table 7:** Genotypes and association analysis of selected candidate genes in boars.

Polymorphism	Boar taint compound (Ln)	Genotype (μ ± S.E.)			Effect (μ ± S.E.)	
		TT	TC	CC	Additive	Dominance
ATP5B T>C	Skatole (µg/g)	4.62±0.34^e^	5.43±0.29^f^	4.32±0.16^e^	0.14±0.17	−0.95±0.31**
		GG	GA	AA		
KRT8 G>A	Skatole (µg/g)	5.25±0.24^e^	4.71±0.30^e^	4.20±0.16^f^	0.52±0.13**	0.01±0.29
		CC	CA	AA		
PGM1 C>A	Skatole (µg/g)	4.95±0.20^a^	4.29±0.19^b^	4.09±0.28^b^	0.42±0.16**	0.23±0.22
		AA	AC	CC		
CYP4A25 A>C	Skatole (µg/g)	4.98±0.33	5.00±0.45	4.29±0.18	0.34±0.19	−0.36±0.46
		GG	GA	AA		
SLC22A7 G>A	Skatole (µg/g)	5.11±0.19^e^	4.73±0.39^e^	4.08±0.16^f^	0.51±0.11**	−0.12±0.39
		CC	CT	TT		
IDH1 C>T	Skatole (µg/g)	5.02±0.22*^c^*	4.50±0.24^cd^	4.06±0.20^d^	0.48±0.12**	0.03±0.27

a.b <0.05;

c.d <0.01;

e.f <0.001;

* p<0.05;

** p<0.01.

## Discussion

### Analysis of RNA-Seq Data

The present study describes the transcriptome profiles of liver tissue from boars with high (HS) and low (LS) skatole content in the backfat by using RNA-Seq. To the best of our knowledge, this study provides the first insight into the transcriptome signature in liver tissues by using RNA-Seq that might be involved in the skatole metabolism. Using the whole transcriptome sequencing technique, we were able to identify the levels of differentially expressed genes and to associate these genes with divergent skatole levels in terms of boar taint. According to the mapping results, the average number of reads was 22.85 million reads and on an average 65.5% of the reads was categorized as mapped reads corresponding to exon reads (Table 1). The proportion of reads mapped to exons of annotated genes was in accordance the previous studies [25], [26], [27] in pig liver transcriptome (60.2–74.9%), but was higher than that reported by Esteva-Codina et al. [28] (44.1%) in porcine male gonad and Gunawan et al. [16] (40.8%–56.63%) in boar livers with divergent androstenone levels. The percentage of annotated reads varies from 15.6% to 74.9% in porcine transcriptome studies [16], [25], [26], [27], [28], [29], supporting our results. The differences between mapping percentages might be due to several factors such as primer biases, GC content, dinucleotide fragmentation sites, independent cell types, laboratory protocols and the selection of reference genome build for annotations [30]. Another factor is that the current reference transcriptome assembly might not cover all the transcribed mRNA [31] and consequently low abundant transcripts or rare alternative splicing isoforms are less likely to be mapped to transcriptome assembly [28]. Illumina sequencing data have been described as replicable with relatively little technical variation [32]. Therefore, the findings of this study clearly demonstrated the power of RNA-Seq and provide further insights into the transcriptome of liver tissue at a finer resolution in skatole divergent boars.

### Differential Gene Expression Analysis

In this study, 448 genes were differentially regulated in liver tissues with divergent skatole levels (Table S1). The top two up regulated gene in the liver sample were *SERPINA12* with log2 fold change 5.81 and *KRT8* with log2 fold change 4.32 (Table 2). *SERPINA12* is identified from visceral adipose tissues of rats, an animal model for obesity and type 2 diabetes [33]. It is reported that SERPINA12 plays an important role in the progression of obesity and insulin resistance [34]. Wada et al. [35] categorized the serine proteases as to be the genes for small hormone-like molecules such as corticosteroid and thyroid hormones. SERPINF2, another member of the same family is involved in 2-aminoacetophenone metabolism which is an important intermediate product of phase 1 skatole metabolism. Notably, the skatole metabolism is divided into two phases: an oxidative phase 1 metabolism and a conjugative phase 2 metabolism. During phase I, skatole is degraded to several intermediate products. Keratins (Ks), the intermediate filaments (IFs) of epithelial cells, constitute the most diversified family of IF proteins with its type I (K9-22) and type II (K1–K8) classes [36]. KRT8/KRT18 IFs can modulate the adhesion, size and cell-cycle progression of hepatic cells, in association with differential plectin/receptor of activated C kinase 1 (RACK1) [37]. The function of highly polymorphic *KRT8* in the skatole metabolism in the liver is not quite clear. However, this gene mapped close to a region on SSC5 affecting skatole and indole levels [38]. The top two down regulated gene in higher skatole group were LOC100737759 with log2 fold change −6.79 and LOC100625674 with log2 fold change −5.88. Till now, it is difficult to identify either the actual gene name or function through orthologous database or BLAST sequence similarity searches. The NCBI database referred LOC100737759 as ‘putative 2-oxo-4-hydroxy-4-carboxy-5-ureidoimidazoline decarboxylase-like’ which might be involved in amino acid metabolism. Similarly, LOC100625674 is referred as ‘cadherin-18-like’ which is a calcium-dependent cell adhesion protein and preferentially contributes in connecting cells. Cadherin-18 is a dominant gene belonging to the remodelling of epithelial adherens junction pathway [39] which is found to be a dominant pathway in our study.

There are similarities between gene expression differences found with RNA-Seq and those reported in previous transcriptome studies in pigs [17], [18], [38], [40]. Similar to the previous studies [41], [42], cytochrome P450 superfamily genes were found to be differentially regulated in skatole catabolism. Phase I skatole metabolism includes mostly oxidative reactions usually performed by the membrane bound cytochrome P450 system [43]. Cytochrome P450 isoenzymes are the main enzymes playing roles in phase 1 skatole metabolism, where skatole is degraded to several intermediate products including such as indole-3-carbinol(I3C), 2-aminoacetophenone (2AAP) and 3-metyloxyindole (3MOI) (details reviewed by [1], [44], [45]). Notably, CYP4A24, CYP4A25 and CYB4B24 were found to be up-regulated in the high skatole Duroc×F2 population in this study which is in agreement with previous results for a Landrace population [8]. Another cytochrome genes family found to be differentially expressed in our transcriptome analysis is cytochrome subunit 5A (COX5A) coding for cytochrome oxidases and previously reported to be associated with skatole levels [7], [13], [46]. Ramos et al. [13] reported that the cytochrome subunit family COX4I1 gene, coding for a subunit of a cytochrome oxidase, is involved in the regulation of porcine skatole metabolism. Additionally, the cytochrome subunit 8C (COX8C) gene, coding for a subunit of cytochrome oxidase is also reported to be involved in the regulation of porcine skatole metabolism [13], [46]. The COX5A gene on SSC7 at position 63.3 Mb maps to an already identified QTL region between 61.5 and 69.6 Mb which is known to affect skatole levels in Duroc and Landrace population. The main enzymes of phase 2 skatole metabolism are UGT (uridine 5′-diphospho-glucuronosyltransferase) and SULT1A1 (sulfotrasferase) [47]. UGT is a family of conjugation enzymes and UGT1A3 is found to be differentially regulated in this study (Table 2). Members of the UGT enzyme family such as UGT1A5 and UGT2A1 are reported to be differentially regulated in porcine transcriptome analysis for androstenone [18]. Different groups of transferases including glutation S transferase omega 2 (GSTO2) and glutathione S-transferase mu 2 (GSTM2) were found to be differentially regulated in this study (Table 2). Glutahione S-transferases (GSTs) are functionally diverse enzymes mostly known to catalyse conjugation reactions of endogenous substances, haem, fatty acids, xenobiotics and products of oxidative processes [48]. In most cases, the effect of conjugation is a decrease of biological activity and increased excretion of these metabolites from the body [49]. During phase 2 metabolism, the water solubility of the skatole metabolism is increased to facilitate excretion via urine [6], [50]. The GSTs, reported to transport different molecules [48], might indicate that the GSTO transports the skatole to the tissues. It could be speculated that GSTO2 might be involved in the excretion of skatole from the porcine body.

Interestingly in this study, four members of SLC family genes such as SLC22A7, SLC25A1 and SLC25A25 were found to be up-regulated and SLC9A4 was found to be down regulated in the high skatole group (Table 2). The solute carrier family (SLC) are important proteins in the regulation of body iron homeostasis and skeletal muscle contains a large proportion of body iron implying the importance of SLC for meat quality traits [51]. The SLC25A1 gene encodes a transporter protein which is responsible for the movement of citrate across the mitochondrial inner membrane [52]. Two members of SLC family (SLC22A13 and SLC22A14) genes were reported to be candidates for taint compounds and sex steroids in pigs [8]. The SLC superfamily is comprised of transporter families involved in the cellular uptake and secretion of endogenous molecules. The substrate panel of SLC22As includes endogenous compounds like tryptophan metabolites and sulphated steroids [53]. Skatole results from a multistep degradation of tryptophan by microbial activity, mainly in the hind gut of the pigs [1], [45]. High concentration of skatole requires a high amount of tryptophan for microbial degradation [1]. Our results show that among the differentially expressed gene in live tissues, genes playing roles in ATP binding (ATP5A1, ATP5B, ATP5D), isocitrate dehydrogenases (IDH1 and IDH3B) and acetyl-CoA (ACSL5, ACOX1) pathways were enriched in functional categories such as in the small molecule biochemistry, protein synthesis, carbohydrate metabolism and energy production (Table 3). In accordance with these results, Ramayo-Caldes et al. [27] also showed that small molecule biochemistry and energy production are members of the enriched GO categories for DEGs in the liver samples.

Pathway analysis of DEGs (Table 4) showed similar patterns with GO analysis and remodelling epithelial of adherens junctions and tricarboxylic (TCA) pathway were found to be the most dominant pathways in this study. Additionally, the mithochondria1 dysfunction and UDP-N-acety1-D-ga1actosamine biosynthesis pathways were found to be enriched in our study (Figure 3). A total of eight genes (*ACTB*, *ARPC3*, *ARPC1A*, *TUBA1A1*, *TUBA1B*, *TUBA1C*, *TUBB2A* and *TUBB4B*) belonging to the remodelling epithelial of adherens junctions pathway are identified in this study and may be involved in the skatole metabolism in the liver (Table 4). The intercellular adherens junctions (AJ) are specialized sub-apical structures that function as principle mediators of cell-cell adhesion [39]. Their assembly-disassembly is dynamic and stringently regulated during tissue morphogenesis and homeostasis [54]. The TCA cycle, found to contain six genes (*DLST*, *IDH3B*, *MDH1*, *MDH2*, *OGDHL* and *SDHD*) is involved in the most important metabolic steps in the mitochondria. The TCA cycle is a catabolic pathway of aerobic respiration and the main source of ATP needed to maintain homeostasis, is produced by oxidation of pyruvate in the TCA cycle [55]. Another over represented canonical pathway in high skatole liver group was the cystein biosynthesis pathway which was previously reported to be responsible for amino acid metabolism in mammalian liver [56]. This pathway includes adenosylhomocysteinase (ACHY), cystathionase (CTH) and FtsJ RNA methyltransferase homolog 1 (FTSJ1) genes (Table 4) which are involved in the amino acid metabolism. These deep sequencing results indicated for the first time the TCA cycle and the cystein biosynthesis to be possibly involved in the metabolism of skatole in porcine liver.

### Differential Exon Expression Analysis

Since an additional important advantage of RNA deep sequencing is the detection of differential exon usage events [25], we used the RNA deep sequencing data to characterize and compare the patterns of differential exon expressions variation in high and low skatole levels. RNA deep sequencing technology provide valuable information regarding alternative and novel splice variants reflecting more complex mechanism of RNA regulation. A previous study by Moe et al. [7] showed that a number of genes involved in RNA processing and translation are differentially expressed between boar taint compounds. This study extends these observations by identifying a number of genes with differential exon expression between high and low skatole level boars. Chen et al. [25] reported that about 18.8% of the annotated genes showed differential exon usage events in pigs with divergent meat quality traits. This study revealed differential level of exon expression for *ATP5B*, *KRT8* and *PGM1* genes in low skatole in comparison to high skatole group suggesting that differential processing of RNA could be associated with the regulation of skatole level.

### Gene Variation Analysis

In addition to the transcriptome quantification, RNA-Seq technology provides valuable information regarding gene polymorphisms which could be directly correlated with the relevant phenotype. Several holistic gene expression analyses have been performed for boar taint compounds by using microarray or Real-Time PCR technology [17], [18], [40]. Our study extends these observations by correlating differentially regulated genes with associated polymorphisms. Gene polymorphisms in the exonic regions might have direct effect on the expression of transcripts and the identified polymorphisms from RNA deep sequencing may give additional insight to the variation in the skatole levels. This study revealed 45 SNPs in 8 highly polymorphic DEGs from liver samples (Table 6). Two highly polymorphic genes *KRT8* and *ATP5B* containing five and three SNPs are mapped close to a region on SSC5 at 18.6 Mb and 23.6 Mb, respectively (Table 6). Several QTL regions incorporating the KRT8 location are reported to affect skatole levels in pigs [38]. On SSC6, we identified 13 polymorphisms in the gene *PGM1* at position 137.1 Mb, six polymorphisms in gene *CYP4A25* at position 152.1 Mb and a set of 7 polymorphisms mapped to the gene *PRDX1* at position 153.2Mb. Several QTL have been identified previously for skatole on SSC6 [9], [13], [57]. On this region of interest, Ramos et al. [13] reported that several SNP markers located close on the region on SSC6 that were significantly associated with skatole levels. Genes coding for cytochrome family have previously been shown to be associated with skatole levels [12], [58] and are mapped on SSC6. These genes are known to be involved in phase I metabolism of skatole [58], [59] implying that these genes could be important positional and functional candidate for boar taint compounds.

Two polymorphism were identified on SSC7 at position 43.8 Mb, mapped to the gene SLC22A7 and a SNP was identified on SSC7 at position 80.5 Mb on the gene DHRS4 (Table 6). Grindflek et al. [8] identified an androstenone related QTL region on SSC7 between region 33.6–41.9 Mb and 80.8–88.3 which is in close proximity to the polymorphisms on gene SLC22A7 and DHRS4, respectively. In addition, a suggestive QTL for skatole is identified on SSC7 in the Yorkhsire pigs [8], overlapping the region harbouring our genes indicating that these markers could be very important for skatole levels. In this study, eight SNPs were identified on *IDH1* gene at position 122.8 Mb on SSC15; yet to the best of our knowledge, no QTL related with skatole trait has been reported in this region. However, a highly significant QTL with a wide confidence interval from 42.5–70.7 Mb is reported on SSC15 in Norwegian Landrace and Duroc affecting the boar taint compounds including androstenone and skatole [8]. Moreover, this region is very rich for several genes involved in cytochrome P450 family and sulfotransferase family activity which are the key enzymes in both of the phases of skatole degradation [1], [8]. Therefore, fine mapping and detailed study of the genes on this region could be interesting.

The selected polymorphisms in genes *ATP5B, KRT8, PGM1, SLC22A7* and *IDH1* were found to be associated with the phenotype skatole level in this study (Table 7). To the best of our knowledge, no study investigated association of the highly polymorphic *ATP5B, KRT8, PGM1, SLC22A7* and *IDH1* genes with boar taint compounds before. Xu et al. [60] reported an association for a SNP in exon 8 (g.75 G>A) in the ATP5B gene with the meat quality traits such as ratio lean to fat, fat meat percentage, intramuscular fat content and intramuscular water content. The ATP5B gene encodes the catalytic subunit of mitochondrial ATP synthesis complex and catalyzes the rate-limiting step of ATP formation in eukaryotic cells [61]. ATP5B probably plays a key role in the porcine skeletal muscle development and may provide further insight into the molecular mechanisms responsible for breed-specific differences in meat quality [60]. However, this study implies that in addition to the meat quality traits, this gene could be an important candidate for boar taint trait. The function of highly polymorphic *KRT8* is associated to pathological processes in liver but the involvement in boar taint is not quite clear. Mutation in KRT8 is reported to be involved in human liver disease [62]. However, this gene maps close to a QTL region on SSC5 affecting skatole and indole levels [38] warranting more studies about this gene and polymorphisms with regards to the boar taint. The gene *PGM1* is involved in glucose metabolism pathway and Lefaucheur et al. [63] found higher expression of genes in glycolytic pathways including this gene in the Large White which is in agreement with high glycolytic and low oxidative metabolism muscle tissues. However, no study has been published so far to unravel the involvement of this gene in boar taint compounds metabolism. The gene *SLC22A7* is involved in the sodium-independent transport and excretion of organic anions and the substrate panel of SLC22As includes important endogenous compounds like tryptophan metabolites and sulphated steroids [53]. Skatole results from a multistep degradation of tryptophan by microbial activity, mainly in the hind gut of the pigs (reviewed by Wesoly and Weiler [1]). Therefore, the marker identified on *SLC22A7* could be a valuable SNP for boar taint. *IDH1* is the most important isocitryte dehydrogenase in the citrate and fatty acid synthesis that is related to energy metabolism and tissue morphology [46]. Energy metabolism is represented by glycolysis and glycogenolyis. It is well established that leaner pigs have a lower ability to synthesize fatty acids combined with greater mobilization, which results in adipose depots with more unsaturated lipids [64]. The pigs with higher metabolism rate such as fatter pigs like Large White and Duroc have higher androstenone and skatole levels than the lean breeds with lower energy metabolism like Pietrain [65]. It is worth to mention that the polymorphisms identified in this study are mostly synonymous and three SNPs from each synonymous and non-synonymous category are validated in this study (Table 6). However, these polymorphisms are suggested to be validated in other porcine populations before considering in selection breeding. The androstenone and skatole levels is correlated (r = 0.27) in the 100 Duroc F2 male pigs used in this study. Grindflek et al. [66] reported r = 0.32–0.36 in 1533 Norwegian Landrace, Strathe et al. [67] found r = 0.37 in 920 Danish intact male, and Windig et al. [68] detected r = 0.37 in 6072 finishing pigs composed of different sire and dam lines. It could be shown that correlation could vary according to the breed and number of animals.

### Conclusion

Here we showed the whole genome expression differences in liver tissues for varying skatole levels in backfat of boars. RNA-Seq provided a high resolution map of transcriptional activities and genetic polymorphisms in this tissue. However, due to incomplete porcine annotations, only around 65.5% of the total reads could be mapped to annotated references. The improvements in pig genome annotations may lead to a better coverage and detailed understanding of genetic and functional variants such as novel transcripts, isoforms, sequence polymorphisms and non-coding RNAs. On the basis of number of the DEGs, our results confirm regulation of transcriptome activity in the liver tissue for skatole degradation. This study proposed candidate genes such as *SERPINA12, KRT8, CYP4A25, COX5A, SLC22A7*, *PRDX1* and *HSD17B2* that might be involved in the liver for skatole metabolism. Importantly, most of the DEGs are functionally related to pathways involved in boar taint and incorporated within the published QTL positions affecting boar taint compounds. Furthermore, various gene polymorphisms were detected in the liver DEGs and their associations are validated with skatole levels. Potential polymorphisms and association were identified for selected mutations in selected DEGs such as *ATP5B, KRT8, PGM1, SLC22A7* and *IDH1*. In addition, differential exon usage analysis of three genes (*ATP5B, KRT8* and *PGM1*) revealed significant differential expression of exons of these genes in the pigs with divergent skatole levels. This transcriptome, polymorphisms and alternative splicing analysis using RNA deep sequencing combined with association analysis revealed potential candidate genes affecting boar taint compound. It is speculated that these polymorphisms could be used as markers for boar taint related traits. However, further validation is required to confirm the effect of these genetic markers in other pig populations.

## Materials and Methods

### Animals and Phenotype

Tissue samples and phenotypes were collected from the Duroc×F_2_ cross animals. F_2_ was created by crossing F_1_ animals (Leicoma × German Landrace) with the Large White pig breed. Duroc×F_2_ boars were on average 116 days old and had on average 90 kg live weight at slaughter. All pigs were slaughtered in a commercial abattoir called Landesanstalt für Schweinezucht - LSZ Boxberg. Slaughterhouse management gave the necessary permissions for the tissue and organ collection. Carcass and meat quality data were collected according to guidelines of the German performance test [69]. As described in Gunawan et al. [16], tissue samples from liver were frozen in liquid nitrogen immediately after slaughter and stored at −80°C until used for RNA extraction. Fat samples were collected from the neck and stored at −20°C until used for skatole measurements. For the quantification of skatole an in-house gas-chromatography/mass spectrometry (GC-MS) method was applied as described previously [70]. Pigs having a fat skatole level less than 0.25 µg/g and greater than 0.25 µg/g were defined as low and high skatole samples, respectively [67], [71]. Six boars were selected from a pool of 100 pigs and the average skatole value for these selected animals were 0.27±0.20 µg/g. RNA was isolated from the liver tissues of 3 pigs with (HS, high skatole group) high (0.45±0.08 µg/g) and 3 pigs with (LS, low skatole group) low levels of skatole (0.09±0.02 µg/g). Notably, these six boars were among the ten boars which have been previously used for androstenone study [16]. Among the ten pigs used in androstenone study, six pigs were found with extremely high and low skatole levels and were considered for this study. There is correlation between the androstenone and skatole levels (r = 0.27) in the 100 Duroc F2 pigs used in this study. Furthermore, these 100 boars were used for association study (Table S5). Total RNA was extracted using RNeasy Mini Kit according to manufacturer’s recommendations (Qiagen). Total RNA was treated using on-column RNase-Free DNase set (Promega) and quantified using a spectrophotometer (NanoDrop, ND8000, Thermo Scientific). RNA quality was assessed using an Agilent 2100 Bioanalyser and RNA Nano 6000 Labchip kit (Agilent Technologies).

### Library Construction and Sequencing

Details of the library construction and sequencing procedures were described previously by Guanwan et al. [16]. The library preparations were sequenced on an Illumina HiSeq 2000 at GATC Biotech AG (Konstanz, Germany). All sequences were analysed using the CASAVA v1.7 (Illumina, USA). As described in Gunawan et al. [16], the deep sequencing data have been deposited in NCBI SRA database and are accessible through GEO series accession number GSE44171 (http://www.ncbi.nlm.nih.gov/geo/query/acc.cgi?acc=GSE44171).

### Genome Reference and Mapping

The first step data analysis was the quality control and filtering step. In this step, PCR primers identified in the raw reads using the FASTQC (http://www.bioinformatics.babraham.ac.uk/projects/fastqc/) quality control application and bad quality sequences with a Phred score of <20 were trimmed off. In this study, the raw reads after quality control were mapped to NCBI Sscrofa10.2 genome build using RNA-seq read mapper TopHat [72]. TopHat is a “splice aware” mapper that uses Bowtie short read aligner [73] for aligning the raw reads to the genomes and further analyses these mapping results for splice junction discovery. After mapping the raw reads to the genome build BEDTools utilities [74] was used to compute the coverage of raw reads to Sscrofa10.2 gene positions for each sample. The expression table thus created was further used in the analysis of differentially expressed genes.

### Differential Gene Expression Analysis

The differential gene expression analysis was designed to contrast the difference in the expression of genes between high and low skatole samples. For differential gene expression analysis with raw count data, the R package DESeq was used [75]. The normalization procedure in DESeq handles the differences in the number of reads in each sample. For this purpose, DESeq first generates a fictitious reference sample, with read counts defined as the geometric mean of all the samples. The reads count for each gene in each sample is divided by this geometric mean to obtain the normalized counts. To model the null distribution of the count data, DESeq follows an error model that uses the negative binomial distribution, with variance and mean linked by local regression. The method controls type-I error and provides good detection power [75]. After analysis using DESeq, DEGs were filtered based on p-adjusted value<0.05 and fold change ≥1.5 [76]. Additionally, the gene expression data was also analyzed using a Generalized Linear Model (GLM) function implemented in DESeq to calculate both within and between group deviances. As a sanity checking and filtration step, we cross matched the results from both analysis (p-adjusted ≤0.05 and fold change ≥1.5 criteria and GLM analysis) and only those genes which appeared to be significant in both the tests (*p*-value ≤0.05), were selected for further analysis [16]. The results of GLM analysis are given in Table S6.

### Differential Exon Expression Analysis

In addition to analyzing the genes that are differentially expressed between high and low skatole samples, the differential expression levels of exons of the same genes between the different phenotype samples were also analysed. For this purpose, we used the R package DEXSeq [24]. The mapped read count data were converted into exon “counting bins” as described in [24]. In the next step, the algorithm normalized sequencing depths for all the samples as described in [75]. In the final step, Generalized Linear Models (GLMs) were employed by the algorithm for each counting bin to test for differential expression between phenotype samples. After the analysis, differentially used exons were filtered using the criteria p-adjusted value<0.05.

### Pathways and Networks Analysis

A list of the DEGs was uploaded into the Ingenuity Pathway Analysis (IPA) software (Ingenuity Systems, www.ingenuity.com) to identify relationships between the genes of interest and to uncover common processes and pathways. Networks of the genes were then algorithmically generated based on their connectivity. The ‘Functional Analysis’ tool of the IPA software was used to identify the biological functions most significant to the data set. Canonical pathway analysis was also utilized to identify the pathways from the IPA library of canonical pathways that were most significant to the data set. We used “Benjamini-Hochberg” multiple testing correction to calculate a *p*-value determining the probability that each biological function or canonical pathway assigned to the data set. The significance levels of p-values obtained for every biological function or canonical pathways were corrected for multiple testing using “Benjamini-Hochberg” correction.

### Gene Variation Analysis

In this analysis, SNP calling was performed on the mapping files generated by TopHat algorithm using samtools mpileup command and associated algorithms [77]. From the variants so generated, only those variants with a minimum Root Mean Square (RMS) mapping quality of 20 and a minimum read depth of 100 were selected for further analysis. In the final step, the selected variants were cross-checked against dbSNP database to identify mutations that are already studied. In order to find out the differentially expressed genes that also harboured sequence polymorphisms, we crosschecked and filtered with the chromosomal positions of these variants against those of DEGs and retained only those variants which mapped to DEG the chromosomal positions. By this way, we were able to isolate a handful of mutations that mapped to DEGs from many thousands of identified potential sequence polymorphisms. In the next step, to understand whether these identified polymorphisms segregate either in only one sample group (high skatole or low skatole group) or in both groups (high and low skatole group), we calculated the read/coverage depth of these polymorphisms in all the samples [16]. The identified SNPs were furthermore classified as synonymous or non-synonymous using the GeneWise software (http://www.ebi.ac.uk/Tools/psa/genewise/last accessed 21.03.2013) by comparing between protein sequence and nucleotides incorporated SNP position [78]. The results of this analysis are detailed in the results section and read coverage for individual samples are given in Table S4.

### Quantitative Real-Time PCR (qRT-PCR) Analysis

For qRT-PCR experiment, total RNA from liver samples were isolated from the 6 boars used for deep sequencing. Additionally, RNA was isolated from the similar tissues of 6 independent boars with divergent skatole level among the remaining 94 boars. cDNA were synthesised by reverse transcription PCR using 2 µg of total RNA, SuperScript II reverse transcriptase (Invitrogen) and oligo(dT)12 primer (Invitrogen). Gene specific primers for the qRT-PCR were designed by using the Primer3 software [79]. Detailed information for primers used in this study was given in Table 8. In each run, the 96-well microtiter plate contained each cDNA sample and no-template control. The qRT-PCR was conducted with the following program: 95°C for 3 min and 40 cycles 95°C for 15 s/60°C for 45 s on the StepOne Plus qPCR system (Applied Biosystem). For each PCR reaction 10 µl iTaqTM SYBR® Green Supermix with Rox PCR core reagents (Bio-Rad), 2 µl of cDNA (50 ng/µl) and an optimized amount of primers were mixed with ddH_2_O to a final reaction volume of 20 µl per well. All samples were analysed twice (technical replication) and the geometric mean of the Ct values were further used for mRNA expression profiling. The geometric mean of two housekeeping genes GAPDH and PPIA were used for normalization of the target genes. The delta Ct (ΔCt) values were calculated as the difference between target gene and geometric mean of the reference genes: (ΔCt = Ct_target_−Ct_housekeeping genes_) as described in Silver et al. [80]. Final results were reported as fold change calculated from delta Ct-values.

**Table 8:** Details of primers used for qRT-PCR analysis and genotyping.

Gene	Reference ID	Primer sequences (5′→3′)	Application	Position*	Enzymes	Annealing temp (°C)	Product size (bp)	RFLP pattern
ATP5B	XM_001929410.2	F:AATCCTTTGATGGTCTCCTT R:AAGATATCATTGCCATCCTG	qRT-PCR	–	–	55	201	
DHRS4	NM_214019	F:TCCTGATGACAAAGGCAGTG R:TGCCTTATCCATCCACAACA	qRT-PCR	–	–	60	108	
GSTO2	XM_001927288.3	F:CACCAGAGTTCCGTTGTCCT R:GTCACGTTCTCCCGATGTTT	qRT-PCR	–	–	55	211	
IDH3B	NM_001044575.2	F:TGTCAGCTTCCAACATGCTA R:TGTGAGGTTGGAGGGAATAA	qRT-PCR	–	–	55	205	
HSD17B2	NM_001167649.1	F:TGCAGAACAGAGGACTGTGG R:GCCATGCATCGTTTGTATTG	qRT-PCR	–	–	54	103	
KRT8	NM_001159615.1	F:ACTTGGACAGGACATCAGAG R:ACTCCAGGCTTCAACTACAG	qRT-PCR	–	–	55	166	
PGM1	XM_003127945.3	F:CCTCCTTCATGTAAAACCTG R:GTTAAGACCAAGGCGTATCA	qRT-PCR	–	–	55	190	
PRDX1	XM_003128039	F:GTCCATGAGAACAACGTCTT R:AAGTGAAACCCTGCTACTGA	qRT-PCR	–	–	55	208	
SDHD	NM_001097516.1	F:GGAGGCTCAGTGTTCTTTGC R:CTGGGTGACAGGTGAATGTG	qRT-PCR	–	–	54	148	
SLC22A7	NM_001044617.1	F:TGGATGGAGTATGGCTGTCA R:GCACTCTTCCTCTCCACGTC	qRT-PCR	–	–	56	139	
PPIA	NM_214353	F: CACAAACGGTTCCCAGTTT R:TGTCCACAGTCAGCAATGGT	qRT-PCR	–	–	58	171	
GAPDH	AF017079	F:ACCCAGAAGACTGTGGATGG R:ACGCCTGCTTCACCACCTTC	qRT-PCR	–	–	60	247	
ATP5B	XM_001929410.2	F:GTAAAGACCTCAGCAACCTG R:TGTTTACTCAGGCCTCTCAT	Genotyping	Exon 7	BciVI	58	167	TT: 113+54 CC: 167
KRT8	NM_001159615.1	F:GGAGGCAAACTTATTGTTGA R:TGAGTCTGGTTGGAGGTTAC	Genotyping	Exon 9	BtsCI	55	170	GG:104+66 AA:170
PGM1	XM_003127945.3	F:TCCTTCTCATAGCTGTCGAT R:CATAATTACCCAGGCTTCAG	Genotyping	Exon 3	AciI	55	172	CC:172 AA:117+55
CYP4A25	XM_003128016.3	F:GCTGACAGATCCACACCTAT R:ACCACCTTCATGTAGTCAGG	Genotyping	Exon 1	HpyCH4V	55	230	AA:123+107 CC:230
SLC22A7	XM_003128039.9	F:AAAGGTTCGACCATGAAATG R: TATGGCAGCTGTCTCTGTGA	Genotyping	Exon 8	BstNI	55	201	GG:201 AA:110+81
IDH1	NM_001159615	F: GGGTTGAGAAGGTTCTGGAT R: CTCCTCGTGGTTCTTCTTCA	Genotyping	Exon 4	HhaI	55	177	CC:98+79 TT:177

* Position according to the coding region in *Sus scrofa.*

### Validation of SNP and Association Study

For the validation of association, six SNPs from the highly polymorphic DEGs as well as the genes known to be involved in the skatole metabolism were selected (Table 7). Genotyping in 100 boars were performed by PCR-RFLP method. In brief, a working solution with a final concentration of 50 ng/µl DNA was prepared and stored at 4°C for further analysis. Polymerase chain reactions (PCR) were performed in a 20 µl volume containing 2 µl of genomic DNA, 1 × PCR buffer (with 1.5 mM MgCl_2_), 0.25 mM of dNTP, 5 pM of each primer and 0.1 U of Taq DNA polymerase (GeneCraft). The PCR product was checked on 1.5% agarose gel (Fischer Scientific Ltd) and digested by using the appropriate restriction enzyme (Table 8). Digested PCR-RFLP products were resolved in 3% agarose gels. Details of GenBank accession numbers, primers sequences, annealing temperature and SNP position used in this study are listed in Table 8. Statistical analyses were performed using SAS 9.2 (SAS Institute Inc, Cary, USA). Effects of slaughter age, husbandry system (pen) as well as genotype on boar taint compound skatole were assessed with a fixed effect model (ANOVA) using PROC GLM. For all models, fixed effects included genotype and pen (group. individual) and age of slaughter was fitted as a covariate for boar taint compound skatole. Due to the skewed nature of skatole, data were transformed with natural logarithm before ANOVA to achieve normality. Least square mean values for the loci genotypes were compared by t-test and p-values were adjusted by the Tukey-Kramer correction [81].

## Supporting Information

Table S1 **Differentially expressed genes in liver from boars with high and low skatole in backfat.**
(XLS)

Table S2 **Differential exon expression in liver from boars with high and low skatole in backfat.**
(XLS)

Table S3 **Polymorphisms in DEGs detected in liver from boars with high and low skatole in backfat.**
(XLS)

Table S4 **Sample read counts for polymorphisms in liver from boars with high and low skatole in backfat.**
(XLS)

Table S5 **Genotype, allele frequencies and the chi-square test of selected SNPs validated using RFLP.**
(DOC)

Table S6 **GLM analysis results for liver DEGs.**
(XLS)
