# Supplementary material for: Identification of the Novel Candidate Genes and Variants in Boar Liver Tissues with Divergent Skatole Levels Using RNA Deep Sequencing
Source: PLoS One. 2013 Aug 26;8(8):e72298. doi: 10.1371/journal.pone.0072298 (PMC3753299; doi:10.1371/journal.pone.0072298)
Supplement: Table S5 — Genotype, allele frequencies and the chi-square test of selected SNPs validated using RFLP. (DOC) [file pone.0072298.s005.doc]

Table Supplementary S5. Genotype, allele frequencies and the chi-square test of selected SNPs validated using RFLP

| Polymorphism position | Chr | Number of boars | Genotype frequency | | |  | Allele frequency | | p-value | Chi-square test |
| --- | --- | --- | --- | --- | --- | --- | --- | --- | --- | --- |
|  |  |  | TT | TC | CC |  | T | C |  | χ^2^ |
| ATP5B g. 23661024 T>C | 5 | 100 | 0.11(11) | 0.17 (17) | 0.72 |  | 0.20 | 0.80 |  | 0.21 |
|  |  |  | GG | GA | AA |  | G | A |  |  |
| KRT8 g.18670859>A | 5 | 100 | 0.22(22) | 0.16(16) | 0.77(77) |  | 0.30 | 0.70 | 0.004 | 0.38 |
|  |  |  | CC | CA | AA |  | C | A |  |  |
| PGM1 g.137174784C>A | 6 | 100 | 0.36(36) | 0.48 (48) | 0.16(16) |  | 0.60 | 0.40 | 0.012 | 0.04 |
|  |  |  | AA(n) | AC(n) | CC(n) |  | A | C |  |  |
| CYP4A25 g.152197351 A>C | 6 | 100 | 0.13(13) | 0.07 (7) | 0.80(80) |  | 0.16 | 0.84 | 0.08 | 0.56 |
|  |  |  | GG | GA | AA |  | G | A |  |  |
| SLC22A7 g.43833000 G>A | 7 | 100 | 0.47(47) | 0.08(8) | 0.45(68) |  | 0.51 | 0.49 | 0.001 | 0.71 |
|  |  |  | CC | CT | TT |  | C | T |  |  |
| IDH1 g.122862530 C>T | 15 | 100 | 0.38(38) | 0.21 (21) | 0.41(41) |  | 0.49 | 0.51 | 0.0001 | 0.34 |
